# Serum Vascular Endothelial Growth Factor Level in Patients with Hepatocellular Carcinoma Undergoing Liver Transplantation: Experience of a Single Western Center

**Published:** 2012-02-01

**Authors:** A. Tan, R. Kim, G. El-Gazzaz, N. Menon, F. Aucejo

**Affiliations:** 1 *BC Cancer Agency-Abbotsford, BC, Canada*; 2 *Department of Solid Tumor Oncology Taussig Cancer Center, Cleveland Clinic Foundation, Cleveland, Ohio, USA*; 3 *Hepatobiliary and Liver Transplant Surgery, Digestive Disease Institute, Cleveland Clinic Foundation, Cleveland, Ohio, USA*; 4 *Department of Gastroenterology and Hepatology, Digestive Disease Institute, Cleveland Clinic Foundation, OH, Cleveland, Ohio, USA*; 5 *Hepatobiliary and Liver Transplant Surgery, Digestive Disease Institute, Cleveland Clinic Foundation, OH*

**Keywords:** Vascular endothelial growth factor, Liver transplantation, Hepatocellular carcinoma

## Abstract

Background: The strongest predictor of tumor relapse after liver transplantation for hepatocellular carcinoma (HCC) is vascular invasion, appreciated only on explant analysis. High serum level of vascular endothelial growth factor (VEGF) is associated with worse outcomes after resection or locoregional therapies but its role in liver transplantation remains undefined.

Objective: We report the first western prospective study exploring serum VEGF in HCC liver transplant patients, correlating pre-operative serum VEGF with poor prognostic histologic features during explant analysis.

Methods: Between May 2008, and June 2010, 75 HCC patients underwent liver transplantation at our institution. Serum VEGF was measured every 3 months until liver transplantation and correlated with histopathologic findings on explant.

Results: There was no significant correlation between pre-transplant serum VEGF levels and tumor burden (median 31.0 pg/mL *vs*. 42.5 pg/mL, p=0.33, for tumors within and beyond the Milan criteria, respectively). Pre-transplant VEGF levels were higher in poorly differentiated tumors compared to well to moderately differentiated tumors, but not statistically significant (median 49.0 pg/mL *vs*. 31.0 pg/mL, p=0.26). Pre-transplant VEGF did not correlate with vascular invasion (median 37.0 pg/mL *vs.* 31.0 pg/mL, p=0.35, in the presence and absence of vascular invasion, respectively).

Conclusion: Pre-operative serum VEGF fails to predict unfavorable histologic HCC features in patients undergoing liver transplantation. Role of serum VEGF in liver transplant HCC patients remains unclear.

## INTRODUCTION

Hepatocellular carcinoma (HCC) represents the fifth most common human malignancy and the third leading cause of cancer death worldwide. When diagnosed, only 30% of patients are candidates for curative therapies [1,2]. This is partly attributed to insufficient surveillance in high risk individuals and lack of effective therapies against hepatitis C virus (HCV) infection, the leading etiology of cirrhosis in western countries [3,4].

Liver transplantation can be curative in selected patients with HCC complicating cirrhosis with five years recurrence free survival >75% for within Milan criteria tumors (single lesion 2–5 cm or ≤3 lesions <3 cm each) and post-transplant relapse rates of 10% [5,6]. This is because smaller tumors are less likely to exhibit foci of microvascular invasion or poor differentiation [7]. When beyond Milan criteria, higher risk of post-transplant recurrence is expected [8,9]. Nevertheless, similar outcomes to within Milan criteria tumors have been reported for larger tumors successfully downstaged by locoregional therapies. Downstaging is therefore a useful predictor of favorable tumor biology when selecting patients for liver transplantation [10,11]. However, in an era of severe organ shortage, molecular biomarkers capable of predicting post-transplant HCC recurrence are needed to improve organ allocation.

Vascular invasion is one of the strongest predictors of tumor relapse after hepatic resection or liver transplantation [12,13]. Unfortunately, this histologic information can only be obtained at the time of surgery and no accurate predictors of microvascular invasion in the pre-transplant setting have been established. Pre-transplant HCC biopsy to assess molecular biomarkers of unfavorable biology has been attempted [14]. However, HCC lesions are often not amenable to percutaneous biopsy. In addition, there is a risk for cancerous cell seeding [15].

Therefore, identification of serum tumor biomarkers capable of predicting microscopic vascular spread with high sensitivity and specificity would be helpful clinically.

Reported serum biomarkers of poor prognosis in patients with HCC include alpha-fetoprotein (AFP), lens culinaris agglutinin (LCA)-reactive AFP percentage of total AFP concentration (AFP-L3%), and protein induced by vitamin K absence (PIVKA) II [16-18]. Specifically, AFP-L3% has been related to progression from moderately to poorly differentiated HCC and PIVKA II has been associated with tumor vascular invasion, whereas progressive levels of AFP have been associated with tumor systemic spread and poor patient outcomes [19-23]. However, many HCCs do not secrete these biomarkers or only do so in modest amounts, impeding their clinical relevance [24,25].

Other studies have proposed identifying circulating tumor cells as a good indicator of vascular dissemination in patients with HCC. However, current techniques to detect circulating malignant cells of HCC origin has poor sensitivity and will need refinement to be clinically relevant [26,27].

Vascular endothelial growth factor (VEGF), a protein that promotes angiogenesis, has an important role in HCC development by fostering tumor cell survival, proliferation and new tumor vessel formation [2]. After hepatic resection for HCC, serum VEGF level has been found to be associated with tumor vascular invasion, metastasis, poor response to locoregional therapies (LRT) and worse patient outcomes [28-31]. However, these experiences have been reported mostly by centers in Asia, and it remains unclear if serum VEGF level has any predictive or prognostic value in western populations, or in liver transplantation for HCC. In this study, we aimed to quantify pre-transplant serum VEGF levels to correlate with tumor vascular invasion during analysis of explanted livers. To the best of our knowledge, this is the first prospective study exploring the role of serum VEGF in HCC patients undergoing liver transplantation in a western medical center.

## PATIENTS AND METHODS

Between May 2008 and June 2010, 75 patients with HCC underwent liver transplantation at our institution. HCC was diagnosed pre-operatively either by characteristic appearance on imaging studies and serum AFP levels concordance with standard criteria [32] or by biopsy in patients with tumor atypical for HCC. Patients with HCC within Milan criteria were eligible for liver transplantation and granted exception MELD points. Patients with HCC beyond Milan criteria without evidence of vascular or extra-hepatic spread who were successfully downstaged or did not exhibit disease progression after locoregional therapies were also eligible for transplantation. 

Peripheral venous blood samples were prospectively collected from patients for serum VEGF every three months until the time of liver transplantation. Blood samples were drawn into a serum separator tube and allowed to clot for 30 min and centrifuged at approximately 1000 g for 15 min, then stored below 20 °C until analysis.

Histopathology examination of the explanted livers was performed by expert pathologists without prior knowledge of serum biomarker levels. The length of the longest axis of HCC was defined as the tumor diameter and the number of lesions was determined by counting only viable nodules. Tumors on the explanted liver were staged according to the Milan criteria (within or out of Milan) and graded into one of three categories according to Edmondson and Steiner system: well, moderately and poorly differentiated. The presence or absence of tumor vascular invasion was also investigated. This study was approved by the our Institutional Review Board. 

Assay of serum VEGF level 

VEGF levels were measured using enzyme-linked immunosorbent assay (ELISA) kit designed to measure human VEGF concentration in the serum, in a centralized lab (Quest Diagnostics Nichols Institute, San Juan Capistrano, California) with no prior knowledge of the clinical data. 

Using this technique, a monoclonal antibody specific for VEGF was precoated onto a microplate. Samples and standards were pipetted into the wells and any VEGF present was bound by the immobilized antibody. After washing away the unbound substances, an enzyme-linked polyclonal antibody specific for VEGF was added to the wells. Following a wash to remove any unbound antibody-enzyme reagent, a substrate solution was added to the wells and color developed in proportion to the amount of VEGF bound in the initial step. The color development was stopped and the intensity of the color was measured. The lab value range established for VEGF is 31 to 86 pg/mL.

Statistical Analysis 

Descriptive statistics were computed for all variables. These include median, 25^th^ and 75^th^ percentiles for continuous variables and frequencies for categorical variables. A univariate analysis was performed to assess factors associated with vascular invasion on explants. Wilcoxon rank sum test was used to evaluate differences in continuous variables, Mantel-Haenszel χ^2^ test was used to compare the number of pre-transplant treatments and number of tumors, and Pearson’s χ^2^ or Fisher’s exact tests were used to compare all other categorical variables. To assess associations between VEGF and gender, HCV, LRT, Milan criteria and poor tumor differentiation, Wilcoxon rank sum test was used. Spearman’s correlation coefficient was used to assess the association between VEGF and age. A p<0.05 was considered statistically significant. SAS ver 9.2 software (The SAS Institute, Cary, NC) was used for all statistical analyses.

## RESULTS

Clinical and histopathologic features 

Seventy-five patients were studied and followed for a median of 10.8 months after OLT. The median age of the cohort was 59 years. Fifty-six (75%) patients were male. HCV infection was the most frequent etiology of cirrhosis affecting 46 (61%) patients. Fifty-four (72%) patients received at least one locoregional treatment prior to liver transplantation. Four (5%) patients experienced HCC recurrence after liver transplant. 

On explant analysis, 57 (76%) patients had HCC within Milan criteria. Microvascular invasion was present in 17 (23%) patients. Eighteen (24%) patients had well differentiated HCC, 50 (67%) had moderately differentiated HCC and seven (9%) had poorly differentiated HCC. Patients’ profiles and clinical characteristics are shown in Table 1.

**Table 1:** Patient Characteristics. Values are presented as n (%) or median (IQR).

**Variables**	**n= 75**
***Demographic and Clinical characteristics***	
Age	59.0 (10.5)
Male	56 (75%)
HCV	46 (61%)
Alive	72 (96%)
Any LRT	54 (72%)
No. of adjuvant Txs prior to OLT	
0	21 (28%)
1	38 (51%)
2–3	16 (21%)
Platelets prior to OLT (x 10^9^/L)	72.5 (58.5)
***Liver Explant***	
Vascular invasion	17 (23%)
Differentiation	
Well	18 (24%)
Moderately	50 (67%)
Poorly	7 (9%)
Within Milan	57 (76%)
Pre-OLT serum VEGF level	47.0 (38)

Patients with vascular invasion tended to have less well differentiated tumors. For instance, all 17 (100%) patients with vascular invasion were found to have moderate to poorly differentiated tumors compared to 40 (69%) of 58 patients without vascular invasion (p=0.08). The Milan status of liver explants significantly correlated with vascular invasion. Eleven of 17 patients with vascular invasion had liver explants pathology which was beyond the Milan criteria *vs*. 15 (26%) of 58 without vascular invasion (p=0.032). The correlation between vascular invasion and liver explant pathology is shown in table 2.

**Table 2:** Vascular invasion and patient characteristics. Values are presented as n (%).

Factor		Vascular invasion(n=17)	No vascular invasion(n=58)	p value
HCC status at diagnosis	Beyond Milan	7 (41)	11 (19)	0.28
	Within Milan	10(59)	47 (81)	
Liver explant differentiation^†^	Well	0 (0)	18 (31)	0.081
	Moderate	14 (82)	36 (62)	
	Poor	3 (18)	4 (7)	
HCC status on liver explant	Beyond Milan	11 (65)	15 (26)	0.032
	Within Milan	6 (35)	43 (74)	

Histopathologic features and serum VEGF levels 

The median (interquartile range (IQR)) serum VEGF level was 47.0 (38.0) pg/mL. No statistical significant relationship was observed between serum VEGF levels and tumor histopathologic features. However, a trend of higher serum VEGF levels was observed in HCC’s with ominous histopathology (poor tumor differentiation, vascular invasion, beyond Milan criteria) (Table 3).

**Table 3:** Pre-liver transplant serumVEGF and histologic characteristics. Values are presented as median (IQR).

**Histologic features**	**VEGF**
Vascular invasion	37.0 (11.0)
No vascular invasion	31.0 (16.1)
p value	0.35
Within Milan	31.0 (12.1)
Beyond Milan	42.5 (34.4)
p value	0.33
Poor differentiation	49.0 (107.1)
Well/Moderate differentiation	31.0 (15.1)
p value	0.26
HCV	34.0 (19.1)
No HCV	31.0 (9.1)
p value	0.27
Locoregional therapy	34 (19.9)
No locoregional therapy	30.9 (17.1)
p value	0.05
Multiple tumors	34.0 (19.9)
1 tumor	30.9 (16.1)
p value	0.11

Those with tumors within Milan criteria had a median (IQR) pre-transplant serum VEGF level of 31.0 (12.1) pg/mL compared to 42.5 (34.4) pg/mL for those with tumors beyond Milan criteria (p=0.33).

Those with poorly differentiated tumors had a median (IQR) pre-transplant VEGF levels of 49.0 (107.1) pg/mL compared to 31.0 (15.1) pg/mL in those with well to moderately differentiated tumors (p=0.26).

Notably, pre- transplant serum VEGF levels had no strong predictive value for tumor vascular invasion with a median (IQR) of 37.0 (19.1) pg/mL *vs*. 31.0 (16.1) pg/mL in HCC patients without vascular invasion (p=0.35).

Serum VEGF levels did not correlate with HCV positivity—the median (IQR) serum VEGF level was 34.0 (19.1) pg/mL in HCV patients *vs*. 31.0 (9.1) in non-HCV patients (p=0.27). There was a borderline association between VEGF and a history of locoregional therapy—the median (IQR) serum VEGF level was 34.0 (19.9) pg/mL in patients with a history of locoregional therapy *vs*. 30.9 (17.1) pg/mL in those without the therapy (p=0.05).

VEGF and parameters of liver function 

In univariate analyses, lower VEGF levels appear to be associated with worse liver function. There was a trend towards lower VEGF levels in patients with existing splenomegaly—the median (IQR) serum VEGF level was 31.0 (16) pg/mL in those with splenectomy *vs*. 37.0 (26.5) in those without splenomegaly (p=0.2). A lower serum VEGF level however significantly correlated with the presence of ascites—the median (IQR) serum VEGF level was 35.0 (16) pg/mL in patients with ascites compared to 31.0 (3) in those without ascites (p=0.03). Worse portal hypertension and liver function, reflected by lower platelet counts and higher serum bilirubin levels, were both associated with lower serum VEGF level (p<0.009, and p<0.023, respectively). A higher international normalized ratio (INR) was associated with lower serum VEGF levels although this correlation was not statistically significant (p=0.14).

## DISCUSSION

HCC is a highly vascular tumor and expresses VEGF [29]. In addition to tissue expression, serum VEGF levels have been associated with disease progression and poor prognosis in various solid malignancies [33,34]. Patients with disseminated cancer appear to have higher serum VEGF levels compared to those with localized disease [35]. Especially for HCC, increased serum VEGF expression is a poor prognostic biomarker in patients undergoing locoregional therapies such as radiofrequency ablation (RFA), transarterial chemoembolization (TACE) [31,32] and hepatic resection [36]. Accordingly, a significant relationship between high circulating VEGF levels and the presence of venous invasion or metastasis has been identified in several studies [37].

Use of inhibitors of VEGF receptors have improved survival in patients with advanced HCC [2]. However, the role of serum VEGF levels or HCC VEGF expression in predicting or monitoring tumor response to antiangiogenic therapies is unknown. A recent study revealed a possible correlation between response to therapy and plasma VEGF levels in patients receiving treatment with bevacizumab for rectal cancer [38].

In this pilot prospective study, we correlated pre-transplant serum VEGF levels with vascular invasion and other histopathologic features in explanted livers from HCC patients undergoing liver transplantation. A previous study from our group revealed that VEGF receptor II expression in HCC was higher than in the surrounding non-cancerous cirrhotic tissue. Interestingly, the expression of such receptor in the arterioles of the non-cancerous cirrhotic tissue was higher in patients who had HCC beyond Milan criteria, suggesting that a stronger pro-angiogenic background would favor progressive carcinogenesis [39]. In an era where anti-angiogenic therapies for HCC are being developed, the role of tissue and serum expression of VEGF might gain relevance. However, the value of VEGF as a diagnostic or prognostic tool for HCC in the setting of liver transplantation remains uncertain.

In our experience, although serum VEGF levels appeared to be higher in patients with vascular invasion compared to those in whom this feature was absent, the difference was not statistically significant (p=0.35). Similarly, pre-transplant serum VEGF levels appeared to be higher in patients with poorly differentiated tumors and tumors beyond the Milan criteria, but these findings were also not statistically significant (p=0.26, p=0.33, respectively) (Table 3). 

These findings are in contrast to various available reports performed in the setting of liver resection and locoregional therapies for HCC, where high serum VEGF levels seemed to correlate strongly with poor prognostic features such as absence of tumor capsule, presence of intrahepatic metastases, microvascular invasion and more advanced stages of disease [29,30,37,40].

Kaseb, *et al* [41], published a recent study on 288 patients with HCC. The plasma levels of insulin-like growth factor-1 (IGF-1) and VEGF were measured. They found that lower plasma IGF-1 and higher plasma VEGF levels significantly correlated with advanced end-stage liver disease and HCC clinicopathologic parameters and poor overall survival; with cut-off values of 26 ng/mL and 450 pg/mL, respectively. They reported a much higher mean serum VEGF levels than what we found in our experience; this may be attributed to the larger tumors in their study, whereas in ours the entire cohort underwent liver transplantation with smaller tumors. 

Most of the available studies have been performed in the setting of liver resection, and not liver transplantation. Accordingly, the severity of liver cirrhosis is likely different with more preserved underlying liver function present in patients eligible for liver resection compared to those undergoing liver transplantation. The extent of underlying cirrhosis may also affect circulating VEGF levels; therefore, the measured level may not reflect tumor VEGF expression. As acute phase reactants, both tissue expression and serum VEGF have an inclination to increase in acute and chronic hepatitis and to decrease in cirrhosis [42,43]. Circulating serum VEGF levels also decrease as histological progression in Child Pugh classification occurs [43].

Moreover, serum VEGF levels are influenced by platelet levels, as VEGF is stored in platelets and VEGF release into the circulation occurs when platelets are activated [29,44]. Patients eligible for transplantation tend to have lower platelet counts for hypersplenism and may have less circulating VEGF level than those with less portal hypertension such as liver resection candidates. Hence, serum VEGF level may not be an accurate indicator of HCC expression of VEGF in patients undergoing liver transplantation. Notably, the median platelet count we found in our study patients was only 72.5 × 10^9^/L.

The confounding effect of platelet’s storage and release of VEGF could be overcome by assessing the plasma levels of VEGF. In addition, VEGF levels may fluctuate based on platelet activation during blood clotting related to processing of serum samples. This effect can be negated by measuring plasma VEGF, which is obtained from anticoagulated blood. In contrast to serum VEGF concentrations, plasma VEGF levels are not affected by the time between blood sampling and analysis. This is important in a clinical setting where blood samples are taken at variable times before analysis [45,46].

The correlation between the severity of liver dysfunction and low serum VEGF levels is supported by our study. We found a median serum VEGF level of 47 pg/mL compared to 245 pg/mL reported by those studies dealing with liver resection [28,29]. Table 4 lists the clinical parameters of liver function studied in our patients. Our patients tended to have low albumin, low platelet counts and ascites, all reflecting higher degrees of liver dysfunction. In univariate analyses, low serum VEGF levels was consistently associated with higher degrees of liver dysfunction as reflected by the presence of ascites (p=0.03), low platelet counts (p=0.009) and high serum bilirubin concentrations (p=0.023). There was also a trend towards a lower serum VEGF level in patients with splenomegaly (p=0.20), and high INR (p=0.14) (Table 5).

**Table 4:** Clinical parameters of liver function in the study

Variables	Median (IQR)
Albumin (g/dL)	3.3 (1)
Prothrombin time(sec)	12.6 (2.1)
INR	1.2 (0.2)
Total bilirubin (mg/dL)	1.3 (1.2)
Platelet counts (x 10^9^/L)	72.5 (58.5)
Ascites*	1 (2)

* 0=No ascites, 1= Mild, 2=Moderate, 3= Massive ascites

**Table 5:** Relationship between studied variables and serum VEGF level

Wilcoxon Rank Sum Test			
Variable		Median (IQR) serum VEGF level	p value
Splenomegaly	Yes	31 (16)	0.20
	No	37 (26.5)	
Ascites	Yes	31 (3)	0.027
	No	35.5 (16)	
Spearman’s correlation coefficients and their 95% confidence interval of variables with serum VEGF level			
Platelet counts		0.30 (0.11to 0.49)	0.009
Serum bilirubin level		0.26 (0.51 to 0.03)	0.023
INR		0.17 (0.39 to 0.04)	0.14

Although finding lower serum VEGF levels in the setting of liver transplantation may be important, the high variability observed in serum VEGF levels made it difficult to define a reliable cut-off value in our study.

Most of the present data on VEGF and HCC originate from Asian countries raising the question of its applicability to western societies. In Asia, HCC is frequently related to hepatitis B virus (HBV) infection, while in Europe and North America, HCV constitutes the most important risk factor for cirrhosis and HCC. Furthermore, HBV can induce HCC in the absence of cirrhosis, while patients with chronic hepatitis C almost always develop HCC in the presence of cirrhosis [47]. The number of HBV-related HCC patients in our study were too small to make any meaningful comparison and it remains unclear if serum VEGF levels are influenced by the etiology of the underlying liver disease. In our study, no significant association between etiology of cirrhosis and serum VEGF was observed.

Our study revealed a trend towards higher serum VEGF levels (p=0.05) in patients receiving locoregional treatments prior to liver transplantation. This has also been reported in other studies, and is likely related to increased tumor VEGF expression from therapy-induced tissue hypoxia [48,49].

The search for accurate predictors of tumor microvascular invasion in patients with HCC needs to be continued. Other promising modalities such as detection of circulating malignant cells may serve as an acceptable approach for early detection of vascular tumor dissemination.

In summary, no statistically significant correlation between serum VEGF and poor prognostic histologic features on explant analysis was noticed. Particularly, no strong association with tumor microvascular invasion was found. The wide variation in serum VEGF levels made it difficult to define a reliable cut-off value. One major limitation of our study was the small study sample size. In addition, the low platelet counts observed in our cohort of cirrhotic patients could have masked HCC VEGF release. Additional investigation is needed to characterize the performance of this and other biomarkers in predicting ominous outcome in patients with HCC.

## Notes

Financial Support: **None.**
